# Spatial patterns of climate change and associated climate hazards in Northwest China

**DOI:** 10.1038/s41598-023-37349-w

**Published:** 2023-06-27

**Authors:** Haojing Chi, Yanhong Wu, Hongxing Zheng, Bing Zhang, Zhonghua Sun, Jiaheng Yan, Yongkang Ren, Linan Guo

**Affiliations:** 1 Key Laboratory of Digital Earth Science, Aerospace Information Research Institute, Chinese Academy of Sciences, Beijing, 100094 China; 2 International Research Center of Big Data for Sustainable Development Goals, Beijing, 100094 China; 3 University of Chinese Academy of Sciences, Beijing, 100049 China; 4 CSIRO Environment, Canberra, ACT 2601 Australia; 5 Network and Information Center of Changjiang River Water Resources Commission, Wuhan, 430010 Hubei China; 6 Institute of Tibetan Plateau Research, Chinese Academy of Sciences, Beijing, 100094 China

**Keywords:** Climate change, Natural hazards

## Abstract

Northwest China (NWC) is experiencing noticeable climate change accompanied with increasing impacts of climate hazards induced by changes in climate extremes. Towards developing climate adaptation strategies to mitigate the negative climatic impacts on both the ecosystem and socioeconomic system of the region, this study investigates systematically the spatial patterns of climate change and the associated climate hazards across NWC based on high resolution reanalysis climate dataset for the period 1979 to 2018. We find that NWC overall is under a warming and wetting transition in climate with change rate of temperature and precipitation around 0.49 °C/10a and 22.8 mm/10a respectively. Characteristics of climate change over the NWC however vary considerably in space. According to significance of long-term trends in both temperature and aridity index for each 0.1° × 0.1° grids, five types of climate change are identified across NWC, including warm-wetting, warm-drying, warm without wetting, wetting without warming and unchanging. The warm-wetting zone accounts for the largest proportion of the region (41%) and mainly locates in the arid or semi-arid northwestern NWC. Our findings show most region of NWC is under impacts of intensifying heatwave and rainstorm due to significant increases in high temperature extremes and precipitation extremes. The warming but without wetting zone is found under a more severe impact of heatwave, particularly for areas near northern Mount. Qinling and northern Loess Plateau. Areas with stronger wetting trend is suffering more from rainstorm.

## Introduction

Climate change, usually characterized by warming, is occurring globally due to natural internal processes or external forcings^1^. As highlighted in the IPCC’s Sixth Assessment Report (SAR), the average temperature across the globe over the past 50 years was the highest in the last two millennia^2^. The global increase in temperature noticeably is accompanied with widespread changes in the climate system represented by various climate variables like precipitation, vapor content and wind^3–5^. Climate change is reshaping the spatial and temporal patterns of global energy and moisture^6–8^.

The warming climate has enhanced and expanded extreme climate events in terms of their intensity, frequency, duration, and range of impact. It is observed that the frequency and intensity of extremely high temperatures have increased in most regions of the globe since 1950^2,9,10^. The frequency and intensity of rainstorm events are also found increased over global terrestrial regions^11,12^. More prominently, the probability of compound extreme events (events in which two or more climatic events occur simultaneously or successively to cause extreme impacts) are becoming more frequent^13–15^. Hydroclimatic hazards (e.g., droughts, floods, wildfires) caused by the extreme events are exacerbating^16,17^.

Climate change however does not perform identically across the globe as the climate system varies with the atmospheric circulation and the underlying surfaces at a regional or local scale. The rate of warming and changes in other climate variables (e.g., precipitation, evapotranspiration) vary considerably over the globe^18^. Some areas in the world are experiencing more dramatic shifts in climate with increasing frequency and intensity of extreme weather events, while other regions may see less changes in climate^19^. In developing region-specific adaptation strategies towards mitigating the negative impacts of climate change, it is therefore imperative to investigate the spatial differences or patterns in climate change together with broad assessments of the associated climate hazards in a region^20,21^, which is invaluable to identify the hotspots of climate change around the world^22^.

Northwest China (NWC) is one of the driest regions in the world among locations at the same latitude and is an ecologically vulnerable area sensitive to climate change^17,23^. Based on ground observations, it has been reported that there is a warm-wetting trend in the region since 1970s^24,25^ and the region is experiencing increasing stress of climatic hazards^26–28^. However, other researchers found significant spatial differences in climate change across the NWC during the late twentieth century, showing a wetting trend in the western NWC while and a drying trend in the eastern NWC^29^. The inconsistency in the finding is largely because of the differences in the temporal span, spatial domain and the data used in different researches. Nonetheless, it is generally recognized that extreme temperature and precipitation events are becoming more frequent, profoundly affecting the vulnerable ecosystems in NWC^30,31^.

This research aims to investigate systematically the spatial pattern of climate change in Northwest China and the associated climate hazards. The spatial pattern of climate change is investigated by categorical mapping, where the essential features of climate change in the region are classified by considering jointly the long-term changes in temperature, precipitation, and potential evapotranspiration. The associated changes in climate hazards measured by extremely high/low temperature and precipitation are then assessed for the region and for each climate change zones respectively. This research bases on the gridded climate reanalysis dataset in NWC, focusing on the period from 1979 to 2018. This research is expected to further extend and expand the understanding of climate change and the related climate hazards in NWC, which could contribute to developing site-specific climate adaptation strategies in mitigating the impacts of climatic hazards.

## Study area and data

### Study area

**Figure 1:**
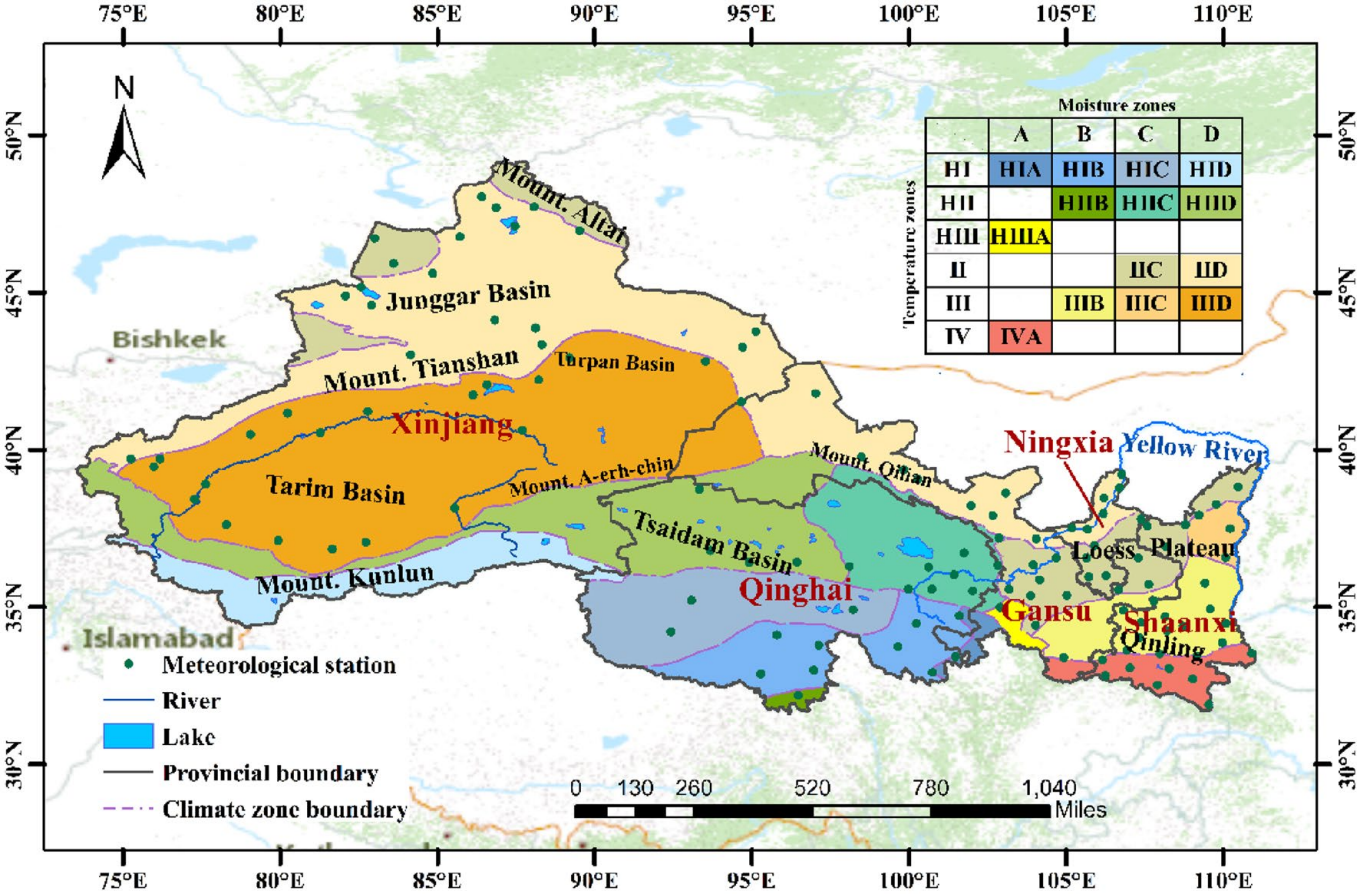
Geomorphologic and climatic regions of Northwest China. HI, HII, HIII, II, III and IV represent plateau sub-frigid, plateau temperate, plateau subtropical, mid-temperate, warm temperate and north subtropical climatic zones, respectively. A, B, C and D represent humid, semi-humid, semi-arid and arid.

Northwest China (NWC) locates at the hinterland of Eurasia and the northeastern Tibetan Plateau (73° 15ʹ –111° 15ʹ E, 31° 32ʹ–49° 10ʹ N) with total area of 3.1 million km^2^. Approximately 34% areas of NWC lies at an altitude above 3000 m, declining from northwest to southeast. Geomorphologically, the region mainly consists of four mountain ranges (i.e., the Mount. Altai in the north, the Mount. Tianshan in the west, the Mount. Kunlun in the south, and the Mount. Qilian in the middle) and three basins (i.e., the Junggar Basin, Tarim Basin, and Tsaidam Basin) between the mountain ranges. The NWC crosses six temperature zones with substantial difference in mean annual temperature between its north and south (Fig. 1). The Tibetan Plateau in the south has lower temperatures due to its high elevation. Climatically, NWC is a dry region in China with regional mean annual precipitation and potential evapotranspiration are around 240.8 mm and 985 mm respectively. Most part of NWC is characterized as arid or semi-arid due to its low precipitation and high evaporation capacity. The region has widespread deserts and the ecosystem within the region overall is vulnerable to climate variation and climate change^32^.

NWC administratively consists of six provinces including Shaanxi, Gansu, Qinghai, Ningxia and Xinjiang. This region is rich in land and mineral resources with population of 103.52 million. The population is mainly concentrated in the Yellow River basin, accounting for about 60% of that in NWC. The economy and population of NWC are growing rapidly. As of 2021, the gross domestic product (GDP) of NWC reached to $106.48 billion. Under a changing climate, the region is experiencing considerable negative impacts from natural disasters. Hydroclimatic hazards like heatwaves, cold waves, droughts and floods pose a great stress on regional socioeconomic system and ecosystem as well.

### Data

The dataset used herein in classifying the regional climate change and assessing the related climate hazards is the China Meteorological Forcing Dataset (CMFD). The CMFD^33,34^ is a gridded reanalysis dataset with spatial resolution of 0.1°. It provides 3-hourly climate data within the China domain for the period 1979–2018, including climate variables like precipitation, temperature, pressure, specific humidity, shortwave radiation, longwave radiation and wind speed. The CMFD dataset was produced by merging conventional meteorological observations from the Chinese Meteorology Agency (CMA) and various existing climate data like Global Land Data Assimilation System (GLDAS)^35^ and the Tropical Rainfall Measuring Mission (TRMM) precipitation data^36^. The dataset shows merits by overcoming the limitation of the sparsely distributed meteorological stations in NWC and has been applied to various research endeavors such as climate model validation and climate zone classification^37^ and tested outperform the other gridded climate datasets for the China domain^38^. For validation of the analysis based on CMDF dataset, the station-based meteorological observations provided by CMA is also used in this study, which includes complete daily measurements of atmospheric pressure, air temperature, precipitation, evaporation, relative humidity, and sunshine duration from 126 stations in NWC for the period 1979 to 2018 (Fig. 1).

## Methods

### Classification of climate change

The spatial patterns of climate change in a region can be represented by categorical mapping that classify the region into different zones according to their essential features of changes in prime climate variables (e.g., temperature, precipitation). The regional climate change classification is crucial to better understand, respond, and plan for the impacts of climate change, and to inform decisions to mitigate and adapt to these impacts. Classification of climate change is different from climate classification^39^. Climate classification^40,41^ generally is based on long-term ‘mean’ performance of the climate system, while climate change classification bases on the change signals in the climate system.

There are several climate change indices available to quantify and track changes in the climate^22,24,42^. These indices provide a way to summarize complex climate data and make it easier to understand and communicate the impacts of climate change. The most widely considered climate indices in detecting regional climate change could be that derived from temperature and precipitation time series, as they provide almost the most perceivable change signals in the climate system. In this research, with specific attention on the hydroclimatic impacts of a changing climate, we consider collectively the long-term trends of temperature (T), precipitation (P) and potential evapotranspiration (PET) in classifying the regional climate change. The PET is a function of various climate variables (like solar radiation, wind speed and temperature as well), reflecting a compounding change in the climate system. The ratio of annual PET to annual precipitation is usually defined as aridity index (AI = PET/P) and is used herein to quantify the trend of wetness across NWC. PET can be estimated by various approaches. Herein, the Penman approach is used and expressed as^43,44^:

1 $$E=\frac{\Delta }{\Delta +\gamma }{Q}_{ne}+\frac{\gamma }{\Delta +\gamma }{f}_{e}\left({u}_{2}\right)\left({e}_{2}^{*}-{e}_{2}\right),$$

where $\Delta$ is the slope of saturated water vapor pressure as a function of air temperature (kPa/°C); $\gamma$ is the hygrometer constant (kPa/°C); ${e}_{2}^{*}$ and ${e}_{2}$ are the saturation vapor pressure and vapor pressure, respectively at 2 m above the surface; ${u}_{2}$ mean wind speed at 2 m above the surface; ${Q}_{ne}$ is the available energy is given by:

2 $${Q}_{ne}=({R}_{n}-G)/{L}_{e},$$

where ${R}_{n}$ is the net incoming radiation (MJ m^–2^ d^–1^); $G$ is the heat flux into the ground (W/m^2^); ${L}_{e}$ is the latent heat of evaporation. The wind function ${f}_{e}({u}_{1})$ in Eq. (1) can be expressed as:

3 $${f}_{e}\left({u}_{1}\right)=0.26\times \left(1+0.54\times {u}_{1}\right).$$

Based on the time series of the temperature and aridity index, the long-term trends in temperature and aridity index are detected by the Mann–Kendall approach^45,46^. The trends are then used to classify the regional climate change. According to the combinations of the trends in temperature and aridity index, basically, regional climate change characteristics is classified into nine groups as shown in Table 1. In Table 1, the significance level of the trends in temperature or aridity index is set to 0.05, which means that the climate variable shows a significant increasing (or decreasing) trend when the Mann–Kendall statistic is above 1.96 (or below − 1.96).

**Table 1:** Criteria of regional climate change classification.

		Temperature		
		Significant increase	No significant change	Significant decrease
Aridity index	Significant increase	Warm-drying (WD)	Drying without warming (ND)	Cool-drying (CD)
	No significant change	Warming without wetting (WN)	Unchanging (NN)	Cooling without wetting (CN)
	Significant decrease	Warm-wetting (WW)	Wetting without warming (NW)	Cool-wetting (CW)

### Indices of climate extremes

In this research, to quantitatively assesses the climatic hazards, we consider six climate extreme indices (CEIs) derived from daily precipitation and temperature for the period 1979 to 2018 (Table 2). These indices are proposed by the World Meteorological Organization (WMO) Expert Team on Climate Change Detection and Indices (ETCCDI) and have been widely used^17,47^. We have adapted slightly the definitions of the indices to make them more applicable in the region, considering both climate conditions in NWC and data availability. As listed in Table 2, ${T1D}_{max}$ and ${T1D}_{min}$ represent the extreme hot and cold weather, while ${HD}_{30}$ and ${FD}_{0}$ represent the durations of heatwave and freezing weather. The ${T1D}_{max}$ and hot days (${HD}_{30}$) are determined by maximum daily temperature time series, while ${T1D}_{min}$ and frost days (${FD}_{0}$) are determined by minimum daily temperature time series. ${P1D}_{max}$ and ${P5D}_{max}$ are indices reflecting extreme high precipitation that may cause severe flash flood or riverine flood.

**Table 2:** Definitions of extreme climate indices.

Indices	Definitions	Units
${T1D}_{max}$	99th quantile of daily maximum temperature in a year	°C
${T1D}_{min}$	1st quantile of daily minimum temperature in a year	°C
${HD}_{30}$	Number of days with daily maximum temperature > 30 °C in a year	day
${FD}_{0}$	Number of days with daily minimum temperature < 0 °C in a year	day
${P1D}_{max}$	Maximum daily precipitation in a year	mm
${P5D}_{max}$	Maximum precipitation of 5 continuous days in a year	mm

### Climate hazard assessment

In the context of climate change impacts, climate hazard refers to the potential occurrence of climate-related physical events or trends that may cause damage and loss^1^. Different regions may have different types and severity of climate hazards depending on the regional geographical conditions. The climate hazards usually are assessed based on indicators related to magnitude and likelihood of occurrence of the climate extremes and their corresponding trends. The indicators of the climate hazards however could vary among regions and researchers^48–50^.

In this study, three types of climate hazards are investigated, including heatwave, cold wave and rainstorm. According to geographical conditions of NWC and data availability, herein, a heatwave event is defined as a consecutive period with daily maximum temperature above 30 °C^51^. The severity of heatwave is then determined by its recurrence rate (${FT}_{30}$) in the region expressed as:

4 $${FT}_{30}=k/n,$$

where, $k$ is number of years with at least one heatwave event, *n* (= 40) is the total number of years in the period 1979–2018. A location with higher recurrence rate of heatwave means having more severe impact of heatwave (Table 3). Meanwhile, a cold wave event is a period with minimum daily temperature below a critical value^52^. It is comparatively hard to set a universal critical value of low temperature in defining cold wave across a region with substantial spatial variation in climate. In this study, we use the long-term mean of the lowest minimum daily temperature ($MT1{D}_{min}$) in a year as a surrogate to assess the severity of cold wave hazard, which is calculated as:

**Table 3:** Criteria of climate hazard assessment.

Climate hazards	Indicators	High severity	Moderate severity	Low severity
Heatwave	$F{T}_{30}$	$\ge 0.2$	0.1–0.2	$<0.1$
Cold wave	$MT1{D}_{min}$	≤ − 30 °C	− 20 to 30 °C	> − 20 °C
Rainstorm	$MP1{D}_{max}$	≥ 20 mm	10–20 mm	< 10 mm

5 $${MT1D}_{\mathrm{min}}=\frac{1}{n}{\sum }_{1}^{n}{T1D}_{min}.$$

The $MT1{D}_{min}$ approximately represents the lowest temperature in a year of a location that will occur once in every 2 years. The smaller the $MT1{D}_{min}$ hence indicates the higher severity of cold wave hazard (Table 3). Similar to the definition in Liao et al.^53^, we herein define the severity of rainstorm hazard in a location according to its long-term mean of maximum daily precipitation in a year ($MP1{D}_{max}$), expressed as:

6 $${MP1D}_{\mathrm{max}}=\frac{1}{n}{\sum }_{1}^{n}{P1D}_{max}.$$

A higher $MP1{D}_{max}$ hence indicates a higher severe impacts of rainstorm hazard (Table 3). As presented in Table 3, each of the three climate hazards is classified into three levels, namely high, medium and low severity according to the criteria, which are derived from literature review and based on climate characteristics in the region.

Considering the trends in the climate extremes (i.e., $T1{D}_{max}$, $T1{D}_{min}$ and $P1{D}_{max}$), each of the climate hazard level is further grouped into two sub-classes, namely intensifying and declining. For instance, a location with high severity of heatwave hazard (${FT}_{30}$ > 0.2) may be experiencing an intensifying (where increasing trend in $T1{D}_{max}$ is detected) or a declining (where decreasing trend in $T1{D}_{max}$ is detected) impact of climate hazard. For simplification, each class of climate hazard is represented by a code consists of three letters and in the format like H-HI. The first letter of the code means type of climate hazard (i.e., H, C and R for heatwave, cold wave and rainstorm respectively), the second letter represents severity of the hazards (H, M and L for high, moderate and low severity respectively) and the last letter means the trend in climate hazard (i.e., I and D for intensifying and declining trend respectively).

## Results

### Spatial patterns of climate change

**Figure 2:**
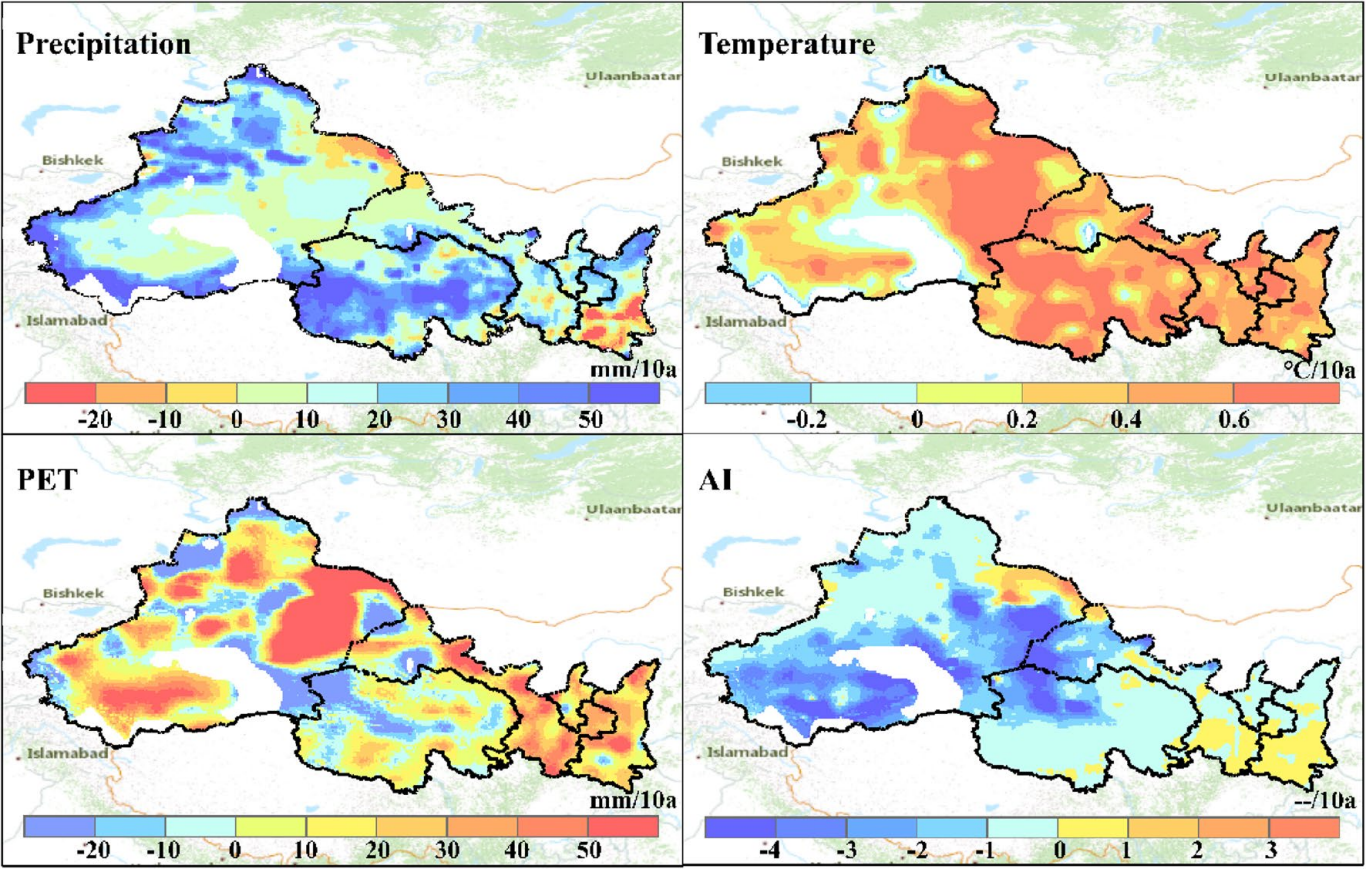
Long-term changes of annual precipitation, temperature, potential evapotranspiration (PET) and aridity index (AI) across NWC for the period 1979–2018.

Figure 2 shows the long-term trends of annual mean temperature, annual precipitation, potential evapotranspiration and aridity index across NWC for the period from 1979 to 2018, basing on the gridded reanalysis dataset (CMFD). Overall, annual mean temperature across NWC is observed to increase at a rate of around 0.49 °C/10a for the period. The most pronounced increase is found at the Junggar Basin and Turpan Basin, which has an increasing rate in temperature higher than 0.6 °C/10a. Largely consistent with the trends in temperature, annual potential evapotranspiration increased over most of NWC at a rate around 20.2 mm/10a. Relatively smaller portion of NWC (e.g., the Tarim Basin and that near the Mount. Altai) is found having a decreased PET. This is because the contributions of temperature to changes in PET could be offset by changes in other climate variables^54^.

**Figure 3:**
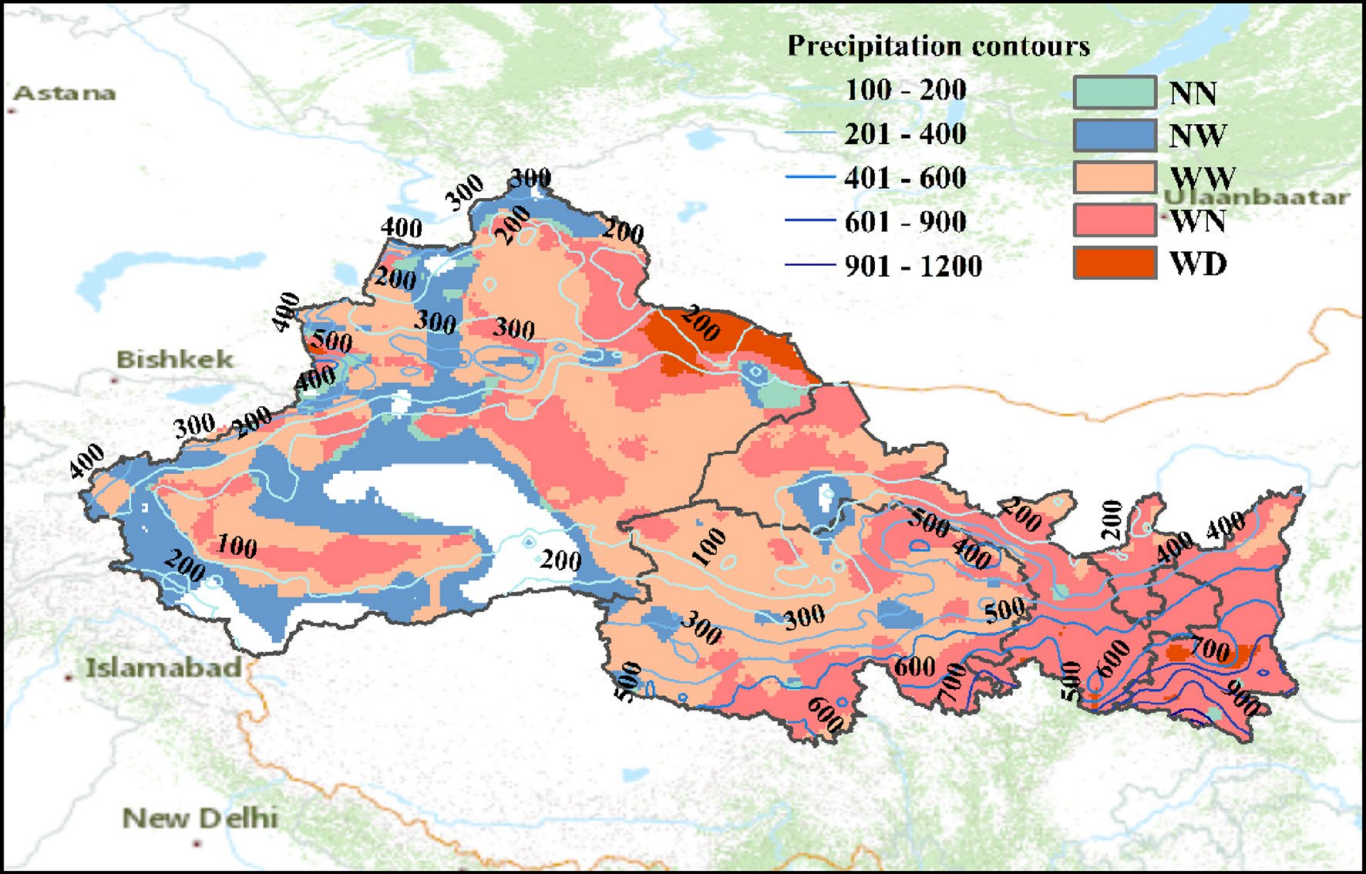
Spatial patterns of climate change in Northwest China. NN, NW, WW, WN and WD represent unchanging, wetting without warming, warm-wetting, warming without wetting and warm-drying, respectively.

Annual precipitation averaged across NWC increases at a rate of around 22.8 mm/10a for the period 1979–2018. The trends in precipitation however show much stronger spatial difference as compared against that of temperature. The arid or semi-arid northwestern NWC (mean annual P below 500 mm) is with an increasing trend in precipitation at a rate around 23.7 mm/10a. Qinghai, Mount. Kunlun and Mount. Altai are found experiencing more significant wetting trend (> 30 mm/10a) than other NWC areas. The southeastern NWC, which is the relatively wetter area in NWC with mean annual P above 800 mm (Fig. 3), however shows a decreasing rate of precipitation up to − 8.8 mm/10a. As a compounding indicator reflecting changes in wetness, aridity index decreases significantly over most of NWC at a rate around − 1.3/10a. The decreasing aridity index suggests increase in precipitation is higher than increase in PET for most part of NWC, resulting an overall wetting trend across the region.

Considering together the long-term trends in annual mean temperature and aridity index for the period 1979–2018, five classes of climate change are identified in NWC. As shown in Fig. 3, in general, there is a wetting trend in the arid or semi-arid area (northwestern NWC) and a more pronounced trend towards warming in the humid or semi-humid area (southeastern NWC). The three overwhelming classes of climate change in NWC (WN, WW and NW) totally account for 95% of the area in NWC and are largely clustered in space. Among the three dominant climate change classes within NWC, the proportion of warm-wetting area is the highest (about 41% of the total area), mainly locates at the arid or semi-arid zone in the western NWC with P < 400 mm. The area belongs to WN (warming without wetting) class mostly locates at the humid or semi-humid zones in the southeastern portion of NWC or near the Mount. Altai, covering about 37% of the total NWC area. The NW (wetting without warming) areas are mainly found in the northern Mount. Kunlun, the central Tarim Basin, and the western Mount. Altai, all within the arid or semi-arid climatic zone. Meanwhile, the other two classes of climate change (WD and NN) account for a relatively small proportion of the total area and spread scattered across NWC, indicating the local geographic effects (e.g., topography, land cover or hydrological conditions) on the climate systems.

### Changes in temperature extremes and associated hazards

**Figure 4:**
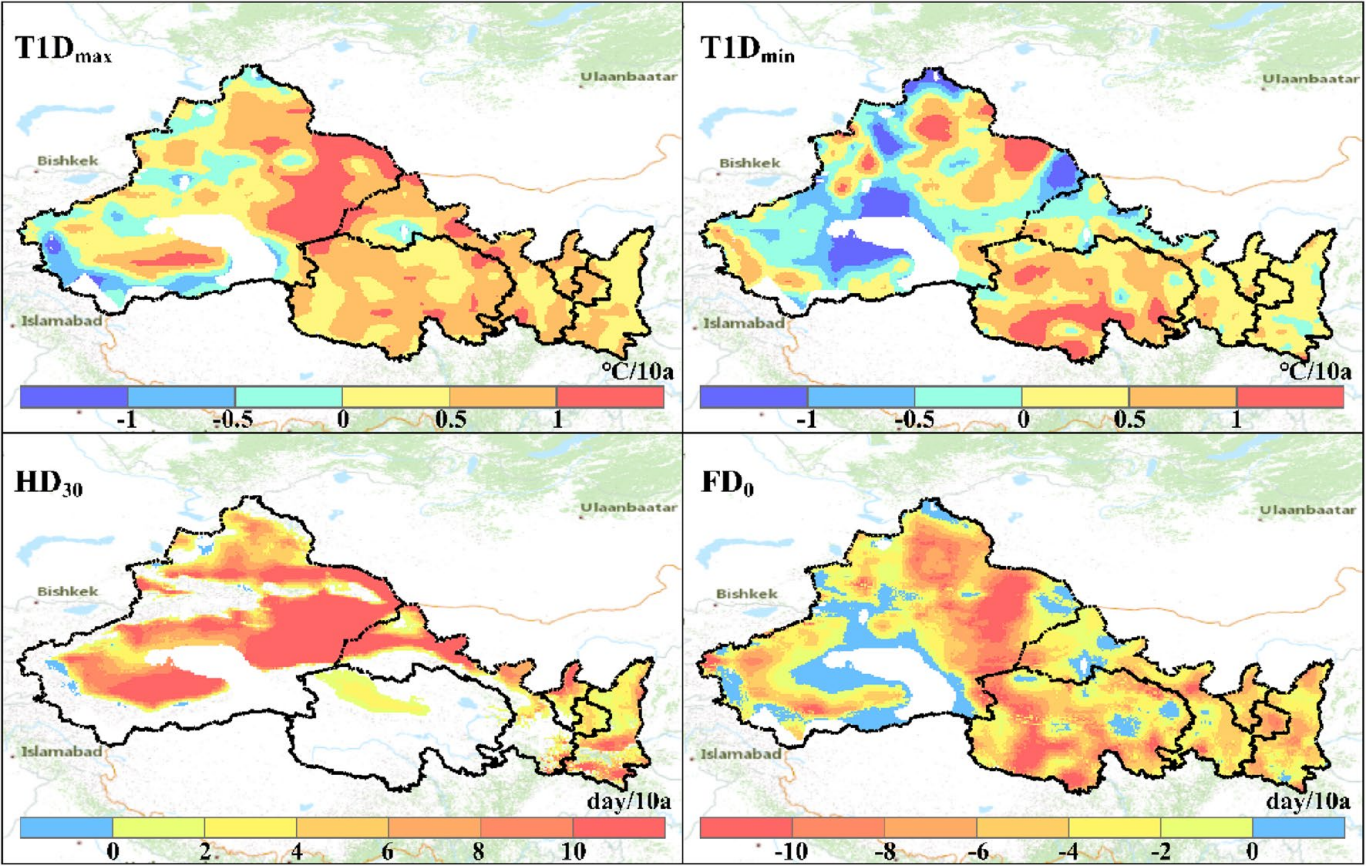
Long-term changes in temperature extremes across Northwest China.

Figure 4 shows the long-term trends detected by the MK approach for the four extreme temperature indices (i.e., ${T1D}_{max}$, ${HD}_{30}$, ${T1D}_{min}$ and ${FD}_{0}$) derived from the gridded reanalysis dataset for the period 1979 to 2018. The intensity and frequency of extremely high temperature demonstrate significant increase trends in most areas. The increasing rate of ${T1D}_{max}$ and ${HD}_{30}$ are 0.5 °C/10a and 4.7 days/10a respectively. Meanwhile, under a warming climate, ${T1D}_{min}$ increases as well but in a lower rate (0.2 °C/10a) than that of ${T1D}_{max}$ and is found within smaller spatial domain (64.8%), along with shortening frost days (− 3.9 days/10a). It is noteworthy that the intensity of both extremely high and low temperatures increases in the Tarim Basin, and the extreme nature of climate are more pronounced in this region.

Changes in the temperature extremes vary across the NWC and among the zones with different characteristics of climate change (Table 4). The warming regions (WW, WD and WN) largely are experiencing higher ${T1D}_{max}$ and ${T1D}_{min}$, longer ${HD}_{30}$ and shorter ${FD}_{30}$. Changes rates of temperature extremes in the WW zone are generally lower than those of the WD zone (Table 4), which implicates cooling effects from wetting. The cooling effects of a wetting climate is more clearly shown in trends of temperature extremes in the NW zone, which contrastively is experiencing lower ${T1D}_{max}$ and ${T1D}_{min}$, and shorter ${FD}_{30}$ though with slightly longer ${HD}_{30}$ (Table 4).

**Table 4:** Long-term trends in temperature extremes for different climate change zones.

Zones	${T1D}_{max}$ (°C/10a)	${T1D}_{min}$ (°C/10a)	${HD}_{30}$ (days/10a)	${FD}_{0}$ (days/10a)
Warm-wetting (WW)	0.6	0.3	9.4	− 5.0
Warm-drying (WD)	1.5	1.0	14.2	− 6.8
Warming without wetting (WN)	0.7	0.2	8.0	− 4.8
Wetting without warming (NW)	− 0.1	− 0.4	6.0	0.9
Unchanging (NN)	0.4	− 0.7	7.5	− 0.2
Entire NWC	0.5	0.2	8.4	− 3.9

**Figure 5:**
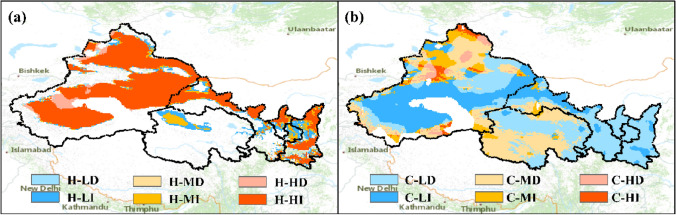
Classification of heatwave (**a**) and cold wave (**b**) impacts across Northwest China. Heatwave and cold wave are noted as H and C in the first letter of the codes. The second letter (H, M or L) represents high, moderate or low severity of the hazard respectively. The last letter (I and D) represents intensifying or declining impacts of climate hazard.

A large proportion of NWC is exposed to the high severity of heatwave impacts (Fig. 5a). Heatwave occurred at least once every 5 years over most NWC. The Tarim Basin, Junggar Basin, Turpan Basin, northern Mount. A-erh-chin, northern Loess Plateau and northern Mount. Qinling are areas with more severe heatwave impacts. The severity of heatwave is found increasing for most of the high severity zone, except for a small portion in western Tarim Basin. For the impact of cold wave, most NWC is subject to low or moderate severity of impacts together with a declining trend (Fig. 5b). The location with high severity of cold wave mainly locates at mountain area (e.g., Mount. Altai, the Mount. Tianshan).

### Climate hazards related to changes in precipitation extremes

**Figure 6:**
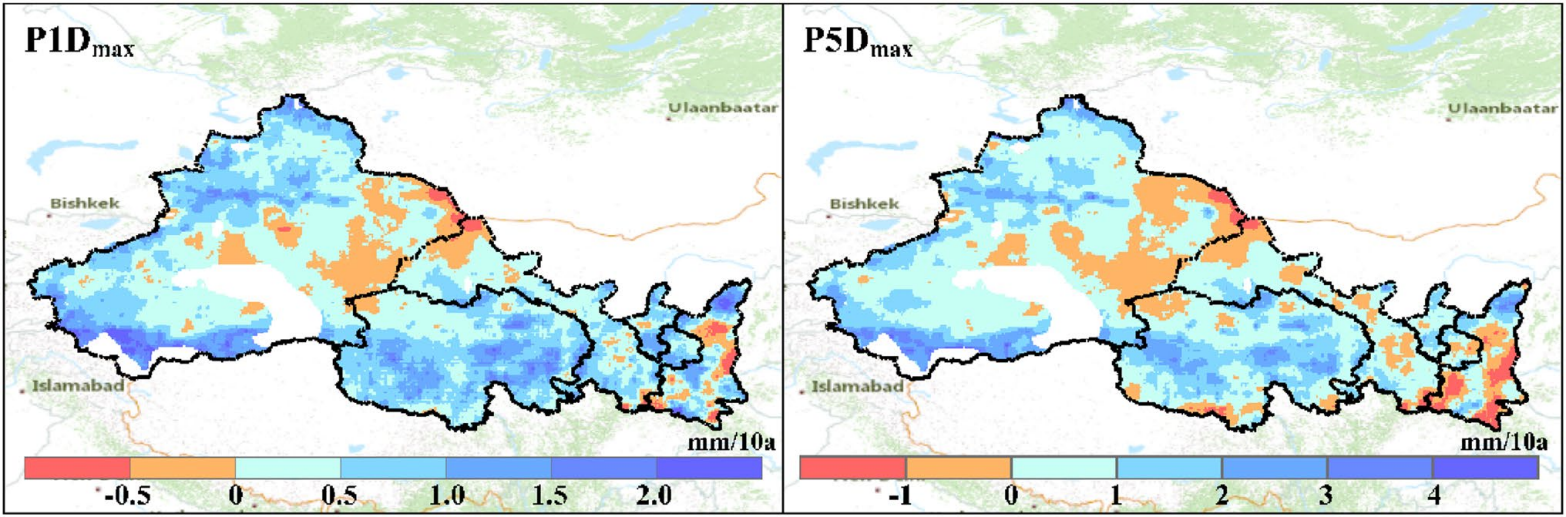
Long-term changes in precipitation extremes across Northwest China.

The long-term trends in precipitation extremes (i.e., $P1{d}_{max}$ and $P5{d}_{max}$) for the period 1979 to 2018 are shown in Fig. 6. There are general upward trends in both two precipitation extremes for most areas of NWC (86.5%). The regionwide mean change rates of $P1{d}_{max}$ and $P5{d}_{max}$ are around 0.5 mm/10a and 0.7 mm/10a respectively. The increases of precipitation intensity are most notably in mountain ranges (e.g., Mount. Altai, Mount. Kunlun and Mount. Tianshan), where the increase rate in annual precipitation could be greater than 2 mm/10a.

The spatial difference of trends in the precipitation extremes are summarized in Table 5 for each climate change zones. The results show that precipitation extremes ($P1{d}_{max}$ and $P5{d}_{max}$) of the wetting zones (WW and NW) increase at higher rates than other zones. The increasing rate of $P1{d}_{max}$ and $P5{d}_{max}$ is the highest in the NW (wetting without warming) zone, reaching to 0.77 mm/10a and 1.25 mm/10a respectively. Meanwhile, the drying zone (WD) is experiencing weakening precipitation extremes.

**Table 5:** Long-term trends in precipitation extremes for different climate change zones.

Zones	$P1{d}_{max}$(mm/10a)	$P5{d}_{max}$(mm/10a)
Warm-wetting (WW)	0.57	0.89
Warm-drying (WD)	− 0.11	− 0.63
Warming without wetting (WN)	0.38	0.26
Wetting without warming (NW)	0.77	1.25
Unchanging (NN)	0.27	0.12
Entire NWC	0.51	0.67

**Figure 7:**
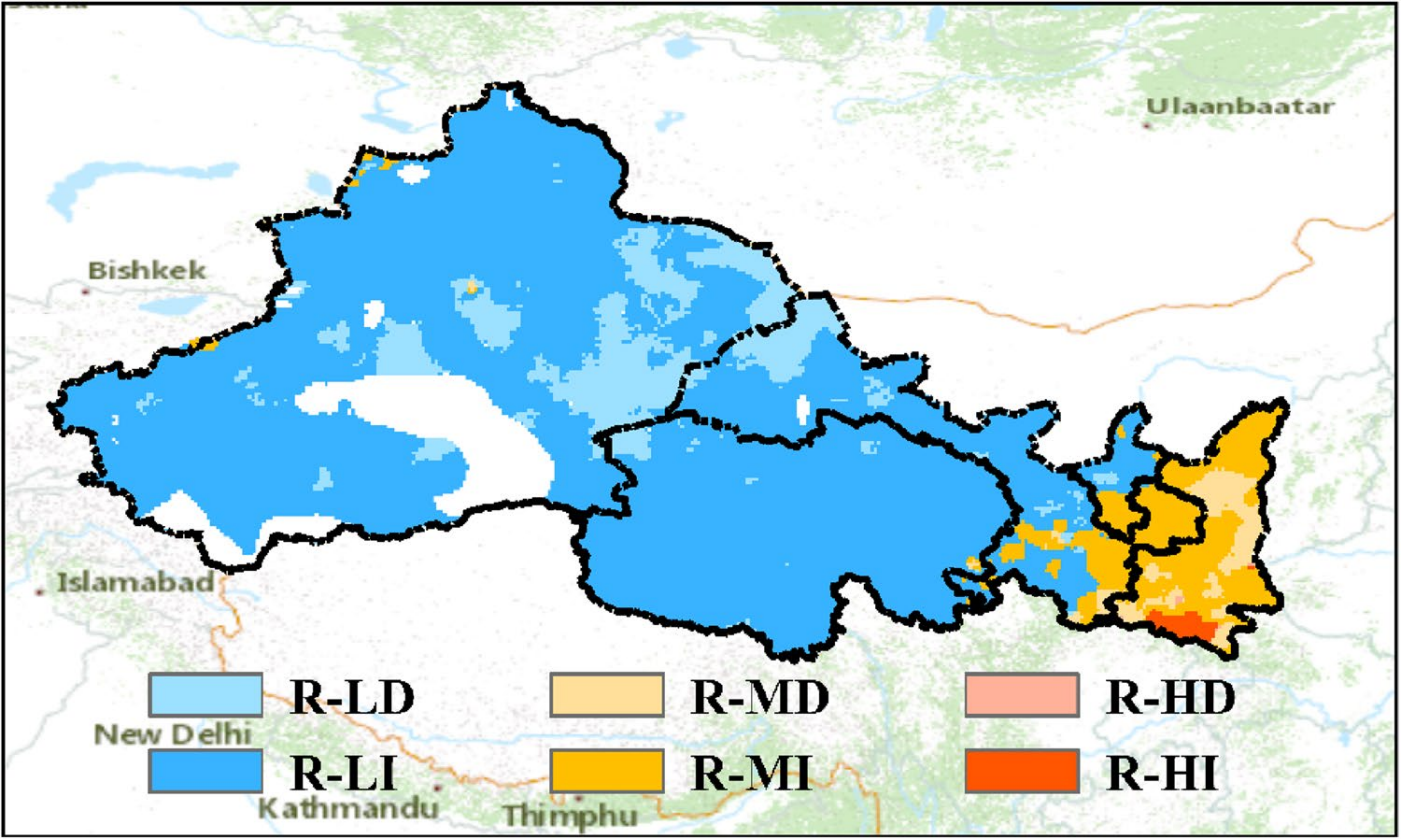
Classification of rainstorm (R) impacts across Northwest China. The second letter (H, M or L) represents high, moderate or low severity of the hazard respectively. The last letter (I and D) represents intensifying or declining impacts of climate hazard.

For climate hazard related to rainstorm, Fig. 7 shows that most of NWC is under low or moderate severity of impact as assessed by its long-term mean of $P1{d}_{max}$. The area with high hazard severity mainly locates in southern Mount. Qinling. This is mainly because that a larger proportion of NWC belongs to arid or semi-arid climate zone and is with low precipitation (Figs. 1, 3). However, the hazard severity is intensifying broadly due to the increase in the precipitation extremes. The area with increasing impacts of rainstorm accounts for 82.8% of NWC. Particularly, it is noted that eastern NWC is with moderate or high severity of rainstorm hazard. The intensifying impacts of rainstorm of in eastern NWC have been demonstrated by disasters observed in recent years^55^.

## Discussions

### Dominant characteristics of climate change in NWC

This study extends and expands upon the previous research on investigating climate change in NWC by using the gridded reanalysis dataset, which covers a larger spatial extent than the previous research that bases on limited ground-based observations. It is found that the warming trend is universal across NWC but the wetting trend limits to the arid or semi-arid area (with mean annual precipitation less than 400 mm) in northwestern NWC. Warm-wetting transition in climate is particularly notable in the arid or semi-arid area in northwestern NWC (e.g., Xinjiang), which is consistent with that reported by Shi et al.^56^. The wetting trend in northwestern NWC however implies only a relative increase in water quantity but not a fundamental change of its dry nature. The eastern NWC, including Shaanxi and eastern Gansu, however is experiencing warming and drying change in climate as also noticed by other researchers^29,57^.

It is important to note that though there is general warm-wetting trend in NWC, substantial climate variation is also observed. For example, in eastern NWC, warming trends have been evident since the early 1960s, but abrupt temperature change is found in the late 1990s, showing declining trend till 2010^58^. For the wetting trend, a decreasing trend followed by an increasing trend was observed since the 1960s, with a cut-off of 1997^59^. It is also worth noting that, wetting trend observed from increasing streamflow or lake expansion in some areas within NWC (e.g., Mount. Tianshan), is not necessarily attributed to increase in precipitation. It however could be because of the increasing glacial meltwater induced by a warmer climate^60^. The wetting trend in areas due to increasing glacial meltwater could be temporary in phase with the shrinking of glaciers^61^.

### Climate hazards in NWC

The NWC is a region vulnerable to climate change and under various impact of climate hazards. Three types of climate hazards in NWC are investigated in this study, including heatwave, cold wave and rainstorm. The heatwave could trigger secondary hazards like drought in NWC, which profoundly affect socioeconomical development in the region. For example, drought accompanied with the persistent high temperature in June 2022 has affected 3227 km^2^ of farmland in Shaanxi Province, directly causing around $35 million economic losses. The severity of cold wave hazard in NWC though is found declining under a warming climate, the warmer winter may delay the fulfillment of chilling requirements and thus lead to later onset of spring phases^62^, which needs further exploration. In NWC, rainstorm could induce secondary natural disasters such as flash floods, landslides and mudslides due to low vegetation cover ratio and loose surface composition in the region.

The climate hazards considered in this study do not cover other types of climate hazards like wildfire, drought and dust storm that could result at substantial negative impact in the region. The results in this study hence have limitations in capturing a full picture of climate hazard in NWC. Further studies are needed to include more climate hazards and other factors of climate risk like exposure and vulnerability^1^. It is worthy pointing out that the definitions of climate hazards could vary among regions and research. For example, in some research, heatwave is more strictly defined as that when maximum and minimum temperatures stay unusually high for 3 or more days^63,64^, and cold wave (or cold snap, cold spell) is defined as a rapid fall in temperature within a 24-h period^65^ or maximum daily temperature dropping below − 3.5 °C^66^. In this study, the severity of climate hazards is assessed according to the magnitude and occurrence of climate extremes and their corresponding trends considering climate characteristics of NWC. The indicators and criteria used in this study may need to adapt accordingly when applied to assess climate hazards of regions with considerable different climate from NWC.

### Uncertainties

**Figure 8:**
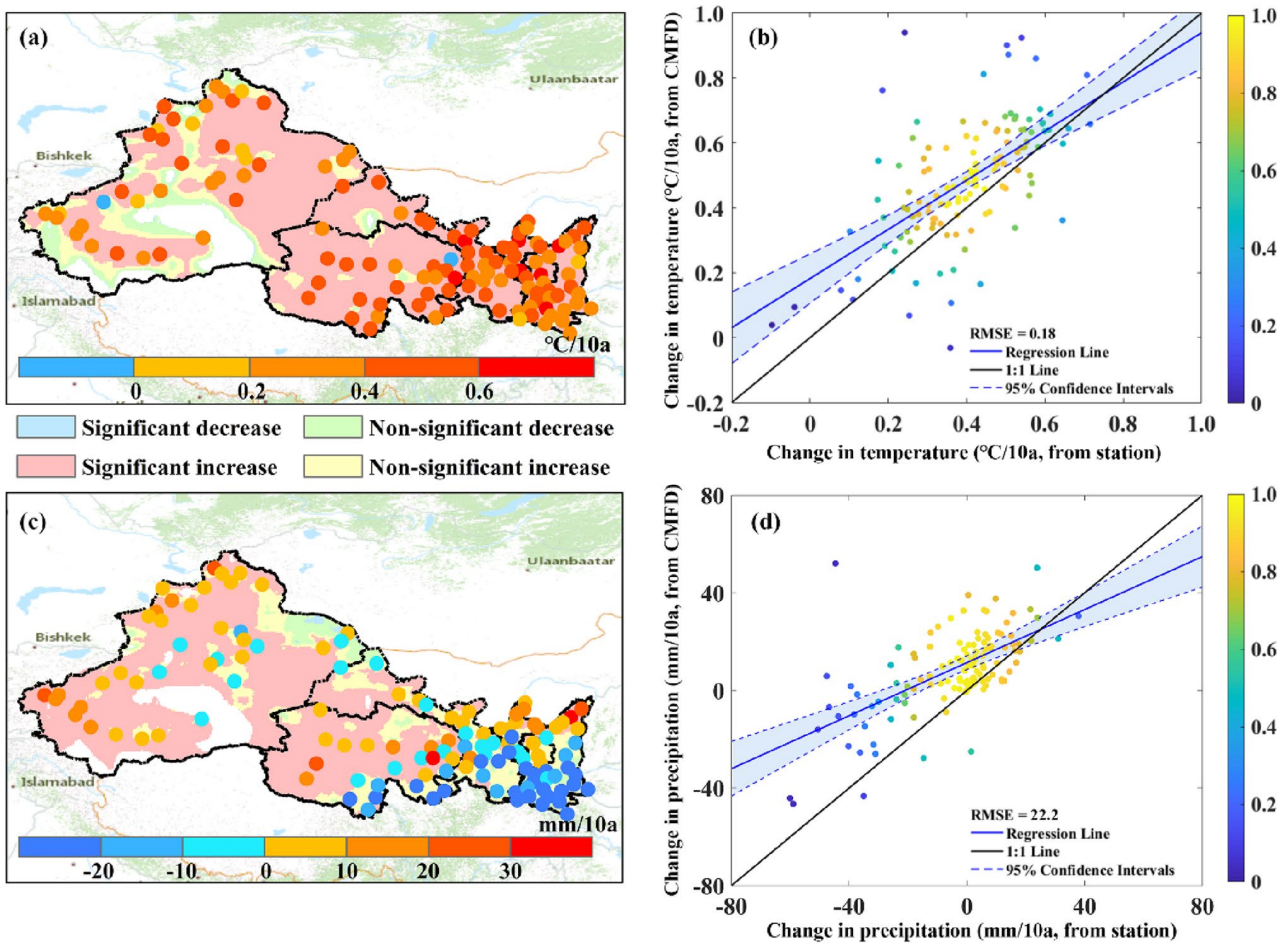
Change rates of temperature (**a,b**) and precipitation (**c,d**) based on reanalysis dataset and station data. The dots in *a* and *c* show the trends derived from station observations, while background layers show the change rates derived from CMFD. $RMSE$ in *b* and *d* represents root mean square error.

The gridded reanalysis climate dataset (CMFD) is used in this study to investigate the spatial patterns of climate change and associated climate hazards in NWC. The findings in this study therefore could have uncertainties inheriting from the dataset even though the dataset has been verified to be of high quality in China^33,67^. As shown in Fig. 8, the long-term change rates of annual precipitation and mean temperature derived from CMFD are largely consistent with those from the station observations. In terms of temperature change, there is high consistency between CMFD and station observation in terms of directions of trend and change rates. For annual precipitation, CMFD aligns closely with station observations in terms of trend direction, but it exhibits a higher rate of change compared to the observations from the stations. Quality of the CMFD dataset should be further investigated or improved in some locations, particularly for area with scarce meteorological stations like southeastern Tarim Basin. For this reason, part of southwestern NWC is not considered in this study due to data reliability (Fig. 8).

## Conclusions

Northwest China (NWC) is an ecologically vulnerable region sensitive to climate change. NWC overall is experiencing noticeable warming and wetting transition in climate during the period 1979–2018. The increasing rate of annual mean temperature and precipitation is around 0.49 °C/10a and 22.8 mm/10a respectively. Though with increasing potential evapotranspiration (20.2 mm/10a), the aridity index decreases significantly over most of NWC at a rate around − 1.3/10a.

Characteristics of climate change over the NWC however vary considerably in space. According to the significance of long-term trends in both temperature and aridity index for each 0.1° × 0.1° grids, five types of climate change are identified across NWC, including warm-wetting, warm-drying, warm without wetting, wetting without warming and unchanging. Warm-wetting area accounts about 41% of total area in NWC, locating mainly at the arid or semi-arid zone in the western NWC. The southeastern NWC and that near the Mount. Altai is found with a warming trend but no significant wetting/drying trend, covering about 37% of the total NWC area.

Although the wetting trend can contribute more water resources for the region, the changing climate in NWC has shown substantial negative impacts on socioeconomic and ecosystem particularly from the intensifying climate hazards related to increasing magnitude and frequency of climate extremes. Our findings show that the high temperature extreme increase significantly over most NWC, resulting more severe impacts of heatwave. The intensity of precipitation extreme is also found widely increased in NWC, potentially leading to more severe impacts of rainstorm which could induce secondary natural disasters such as flash floods, landslides, and mudslides.

Climate hazards in NWC are not limited to the three types (heatwave, cold wave and rainstorm) investigated in this study. For a more complete picture of climate risk in NWC under a changing climate, further studies are needed to assess the severity of other climate hazards (e.g., hail, sandstorm or wildfire, etc.) and include other factors of climate risk (e.g., exposure and vulnerability). In the future, it is also important to explore further the drivers dominating the spatial patterns of climate change in Northwest China.

## Data Availability

The datasets used and/or analysed during the current study are available from the corresponding author on reasonable request.
